# *OAZ1* knockdown enhances viability and inhibits *ER* and *LHR* transcriptions of granulosa cells in geese

**DOI:** 10.1371/journal.pone.0175016

**Published:** 2017-03-31

**Authors:** Bo Kang, Dongmei Jiang, Rong Ma, Hui He, Zhixin Yi, Ziyu Chen

**Affiliations:** 1 College of Animal Science and Technology, Sichuan Agricultural University, Chengdu, Sichuan Province, People’s Republic of China; 2 Farm Animal Genetic Resources Exploration and Innovation Key Laboratory of Sichuan Province, Sichuan Agricultural University, Chengdu, Sichuan Province, People’s Republic of China; University of Toronto, CANADA

## Abstract

An increasing number of studies suggest that ornithine decarboxylase antizyme 1 (OAZ1), which is regarded as a tumor suppressor gene, regulates follicular development, ovulation, and steroidogenesis. The granulosa cells in the ovary play a critical role in these ovarian functions. However, the action of OAZ1 mediating physiological functions of granulosa cells is obscure. *OAZ1* knockdown in granulosa cells of geese was carried out in the current study. The effect of *OAZ1* knockdown on polyamine metabolism, cell proliferation, apoptosis, and hormone receptor transcription of primary granulosa cells in geese was measured. The viability of granulosa cells transfected with the shRNA *OAZ1* at 48 h was significantly higher than the control (*p<0*.*05*). The level of putrescine and spermidine in granulosa cells down-regulating *OAZ1* was 7.04- and 2.11- fold higher compared with the control, respectively (*p<0*.*05*). The *CCND1*, *SMAD1*, and *BCL-2* mRNA expression levels in granulosa cells down-regulating *OAZ1* were each significantly higher than the control, respectively (*p<0*.*05*), whereas the *PCNA* and *CASPASE 3* expression levels were significantly lower than the control (*p<0*.*05*). The estradiol concentration, *ER* and *LHR* mRNA expression levels were significantly lower in granulosa cells down-regulating *OAZ1* compared with the control (*p<0*.*05*). Taken together, our results indicated that *OAZ1* knockdown elevated the putrescine and spermidine contents and enhanced granulosa cell viability and inhibited *ER* and *LHR* transcriptions of granulosa cells in geese.

## Introduction

Ornithine decarboxylase antizymes (OAZs) bind to ornithine decarboxylase (ODC), which in turn enhance the degradation of the enzyme protein by the 26S proteasome and also inhibit intracellular polyamine influx [1–3]. Thus, OAZs are considered as negative regulators of intracellular polyamines. Polyamines are essential for cellular processes such as cell growth and proliferation, and hence play important roles in reproduction [4, 5]. Among four types of OAZs described to date [6], the prototype and the most highly investigated form is OAZ1, which is expressed ubiquitously at significantly higher levels and is believed to be the predominant factor in the regulation of ODC [7, 8].

The essential role of OAZ1 in inhibiting ODC and polyamine uptake suggests that OAZ1 is a negative regulator of cell proliferation and tumor development [9–11]. Recent studies have indicated that OAZ1 also prevents centrosome abnormalities and facilitates DNA double-strand break repairs [12, 13]. Fong *et al*. reported that *OAZ1* overexpression reduced the forestomach cell proliferation and increased apoptosis in mice with forestomach carcinogenesis [14]. Further, *OAZ1* overexpression has been shown to have a tumor-suppressive effect in C57BL/6 and DBA/2 mice [15]. In addition to accelerate the degradation of ODC, an increasing number of studies indicate that OAZ1 also binds to and accelerates the degradation of other proteins shown to regulate cell proliferation, such as AURKA, CCND1, and SMAD1 [16–18].

The goose is one of the most important waterfowl species and is also a vital component in the fast-growing poultry industry of China [19, 20]. The study to improve goose laying performance is very important for poultry industry development. Our previous study suggested that increased *OAZ1* expression might disrupt polyamine homeostasis by inhibiting ODC activity and suppress follicular development in geese [21]. Recently, studies from our and others laboratories indicate that OAZ1 regulates the ovarian and follicular development and ovulation by mediating intracellular polyamine homeostasis in the ovary [21–23]. Granulosa cells play a critical role in ovarian functions such as follicular development, ovulation, and steroidogenesis in both mammals and birds. To date, and to our knowledge, studies on OAZ1 regulating ovarian functions, particularly granulosa cells, are scarce. The action of OAZ1 mediating physiological functions of granulosa cells is obscure. We therefore undertook studies using short hairpin RNA (shRNA) targeted to *OAZ1*, with the aim of determining the importance of *OAZ1* in polyamine metabolism, cell proliferation, apoptosis, and hormone responsiveness of primary granulosa cells in geese. The results indicated that *OAZ1* knockdown elevated the putrescine and spermidine contents and enhanced granulosa cell viability and inhibited estrogen receptor (ER) and luteinizing hormone receptor (LHR) transcriptions of granulosa cells in geese.

## Materials and methods

### Ethics statement

All animal experiments of this study were approved by the Animal Care and Use Committee of the Sichuan Agricultural University (Chengdu, China), in order to ensure compliance with international guidelines for animal welfare.

### Animals, primary granulosa cell collection and culture

The Sichuan white geese (*Anser cygnoides*) used in this study were from the Experimental Farm of Waterfowl Breeding in Sichuan Agricultural University (Ya’an, China). The animals were kept under same environmental conditions and provided *ad libitum* water and locally available commercial feed. The geese were exposed to natural lighting and temperature. Adult female geese during the egg-laying stage were killed by exsanguination to obtain the whole ovary under anesthesia with 3% isofulurane (35 mg/kg body weight). The primary granulosa cells were collected as the method described by Gilbert *et al*. [24]. Granulosa cells were cultured in a humidified incubator at 37°C and 5% CO_2_ in a Dulbecco's Modified Eagle Medium/Nutrient Mixture F-12 (DMEM/F-12, Thermo Fisher scientific, Shanghai, China) medium supplemented with 3% fetal bovine serum (FBS) and 100 U/ml penicillin/streptomycin to a concentration of 1 × 10^5^ cells/ml.

### Granulosa cell viability assay

Granulosa cell viability was determined using a 3-(4, 5-Dimethyl-2-thiazolyl)-2,5-diphenyl-2H-tetrazolium bromide (MTT) assay. Granulosa cells were plated at a density of 3–5×10^3^ cells per well in a 96-well plate. Viable cells were stained with MTT solution (0.1 mg/ml, 4 h). Formazan crystals were dissolved by 100 μl dimethylsulfoxide (DMSO). Absorbance was measured at 570 nm using a spectrophotometer (Thermo Fisher scientific, Vantaa, Finlan). Each analysis was performed using three replicate wells.

### shRNA *OAZ1* construction and transfection

Plasmid DNA encoding green fluorescent protein (GFP) and shRNA *OAZ1* vector was constructed from Pentr/U6/shRNA/GFP vector (BGI, Shenzhen, China). The targeted *OAZ1* sequence (GenBank accession number KC845302) was 5′-GCGGATACTCAACAGTCACTG-3′ using the specific primers (forward: 5′-CACCGCGGATACTCAACAGTCACTGCGAACAGTGACTGTTGAGTATCCG-3′ and reverse: 5′-AAAAGCGGATACTCAACAGTCACTGTTCGCAGTGACTGTTGAGTATCCGC-3′). Ligation products with shRNA *OAZ1* were transformed into *Escherichia coli* DH5a competent cells, and verified by sequencing. For transfection, 1.0 μg of shRNA *OAZ1* plasmid, 3.0 μl X-tremeGENE™ HP DNA Transfection Reagent (Roche, Shanghai, China), and up to 100 μl DMEM/F-12 medium were applied per well of 12-well culture plates and co-incubated overnight. The level of *OAZ1* mRNA expression was detected using quantitative real-time PCR (qPCR) as detailed below. Granulosa cells and culture medium were harvested at 24, 48, and 72 h after transfection for the analyses. Each group had three replicates, and the same treatment was repeated in triplicate.

### Total RNA extraction and qPCR

Total RNA was extracted from granulosa cells using the RNAiso Plus kit (Takara, Dalian, China) following the manufacturer’s protocol. Reverse transcription to obtain cDNA was performed using a PrimeScript™ RT reagent kit with a gDNA Eraser (Takara). Primers used in this experiment were synthesized in BGI Company (Shenzhen, China) (Table 1). qPCR detection and expression analysis of genes was then carried out using the iQ SYBR Green Supermix kit (Bio-Rad Laboratories, CA, USA). Threshold and threshold cycle (Ct) values were determined automatically by the CFX Manager^TM^ Software (Bio-Rad Laboratories) using default parameters. The comparative cycle threshold (2^-ΔΔCt^ method) was used to analyze the expression levels of genes examined in this study. The abundance of each gene transcripts was normalized by *GAPDH* gene expression levels and expressed as arbitrary units (AU). The relative quantization of gene expression was performed in three replicates for each sample.

**Table 1:** Oligonucleotide PCR primer sets used in this study.

Primers	Sequence (5’ - 3’)	Size	Tm
*OAZ1*	F: CAGGTGGGCGAGGGAATAGT	142 bp	65°C
	R: GCATCTGTAAGCCTTGACTGGAC		
*OAZ2*	F: AAGCCTCATGTTGTCCACTTC	142 bp	65°C
	R: GTGCTGATAACCCTTCTTTGC		
*AZIN*	F: GCTCTTACTGCACATTGCCACA	180 bp	58°C
	R: TGAATGTACGTTTGCAGTTCCTTG		
*ODC*	F: TGTATCTGCTTGACATTGGTGGTG	146 bp	60°C
	R: CAGGAAGATACTATGTCGCATCAGC		
*SAMDC*	F: GCTTGACCCAGTAGTTATGGACCA	180 bp	58°C
	R: TGAATAGTCCAGTAAGTTCCATCCG		
*SPDS*	F: TCTGCTGCCAAGGTGAGTGC	111 bp	55°C
	R: AGGGATGGTGCAATAGGCGTA		
*SPMS*	F: GTGCTGATCCTTGGAGGTGGT	110 bp	55°C
	R: TTACACCCGTCGATCACCATT		
*SMO*	F: GGCATCAATACCACCGACAAG	116 bp	55°C
	R: TAAGTCAAGCTCTCGCTCTCCG		
*SSAT*	F: CACCCTTTCTACCACTGTCTG	173 bp	58°C
	R: CCAATGCCAAGTCCTCTGT		
*APAO*	F: GAGTTTGAGCAACCCTTCTGG	143 bp	58°C
	R: TGGCTGGAGGACCACAAA		
*CCND1*	F: CTGCTTCGTCCTCTACAGTCTTTG	163 bp	55°C
	R: TTCTTGGCAGGCTCGTAAACA		
*AURKA*	ATCATACTGTCATTCAAAGAGCGTG	169 bp	55°C
	CATTTCAGGAGGCAAGTAGTCAAG		
*PCNA*	F: AGAAATGAATGAGCCAGTCCAGC	178 bp	55°C
	R: TTCAATCTTTGGAGCCAGGTAGT		
*BCL-2*	F: GATGCCTTCGTGGAGTTGTATG	98 bp	60°C
	R: GCTCCCACCAGAACCAAAC		
*SMAD1*	F: CCGCTTGTAGTGGTAAGGATTGA	146 bp	55°C
	R: AATTACATGCGGCAGCCCTT		
*BAX*	F: CCACAAGCAAGCAAAGAGCC	162 bp	58°C
	R: CTGTAATGGCAACTGTAAATGCTTC		
*CASPASE 8*	F: GGTGTCGCAGTTCAGGTA	127 bp	57°C
	R: CATTGTAGTTTCAGGGCTT		
*CASPASE 9*	F: TTCCAGGCTCTGTCGGGTAA	150 bp	64°C
	R: GTCCAGCGTTTCCACATACCA		
*CASPASE 3*	F: CTGGTATTGAGGCAGACAGTGG	158 bp	60°C
	R: CAGCACCCTACACAGAGACTGAA		
*ER*	F: ACCCAAACAGACCATTCAACGAA	187 bp	56°C
	R: CGCCAGACTAAGCCAATCATCAG		
*FSHR*	F: TCCTGTGCTAACCCTTTCCTCTA	207 bp	59°C
	R: AACCAGTGAATAAATAGTCCCATC		
*LHR*	F: GTAACACTGGAATAAGGGAAT	191 bp	53°C
	R: GAAGGCTTGACTGTGGATA		
*GAPDH*	F: GTGGTGCAAGAGGCATTGCTGAC	86 bp	55°C
	R: GCTGATGCTCCCATGTTCGTGAT		

### Polyamine contents measurement

Putrescine, spermidine, and spermine contents in granulosa cells were determined by high performance liquid chromatography (HPLC) analysis with an Agilent 1100 Series system (Agilent Technologies, CA, USA) following a benzoylation procedure. The separation was achieved on a chromatographic column Chromstar ^TM^ C18 (5 μm, 4.6 × 250 mm, Agilent Technologies) in an HPLC system using a fluorescence detector set excitation wavelength at 229 nm. The proportion of mobile phase A (methyl alcohol) and B (water) was 62:38 (v/v). The isocratic elution was performed as follows: 17 min, 62% mobile phase A. The column temperature was maintained at 25°C, the flow rate was at 1 ml/min, and the injection volume was 20 μl. Results were compared to the internal standard (1, 6-hexanediamine) and the standard curves for putrescine, spermidine and spermine standards (Sigma-Aldrich, Shanghai, China). The inter-assay coefficients of variation were <10%.

### Hormone assays

Levels of follicle-stimulating hormone (FSH), LH and estradiol in the culture medium for granulosa cells were determined by using a Goose hormone ELISA kit (Bangyi, Shanghai, China). Briefly, the 50 μl standard was added to the standard wells, and 10 μl of the measuring sample and 40 μl of the sample diluent were added to the measuring sample wells. Then, 100 μl of horseradish peroxidase-conjugate reagent was added, and the wells covered with microplate sealers and incubated for 60 min at 37°C. Each well was aspirated and washed five times with washing buffer. Next, 50 μl of chromogen solution A and 50 μl of chromogen solution B were added to each well. Wells were gently mixed and incubated for 15 min at 37°C, and protected from light. Finally, 50 μl stopping solution was added, and hormone levels were determined by absorbance at 450 nm within 15 min using a spectrophotometer (Thermo Fisher Scientific). The standard curve indicated a direct relationship between optical density and hormone content.

### Statistical analyses

The data were analyzed by a one-way analysis of variance using SAS 9.1 statistical software (SAS Institute Inc., NC, USA). Statistically significant results were further analyzed by Duncan’s multiple range test. The data were presented as the mean ±SEM. A value of *p*<0.05 was considered significant.

## Results

### *OAZ1* knockdown enhanced the viability of granulosa cells

**Fig 1:**
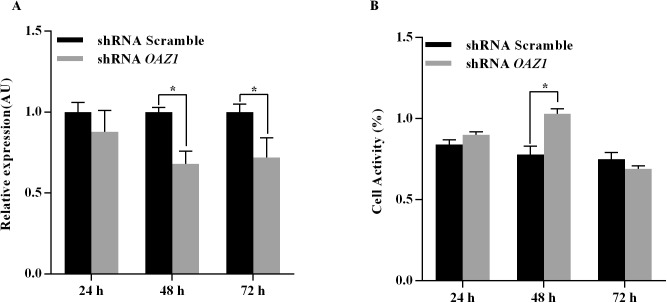
Characteristics of granulosa cells in geese 24 h, 48 h and 72 h after shRNA *OAZ1* transfection. A: The *OAZ1* mRNA expression level in granulosa cells. B: The activity of granulosa cells.

Geese primary granulosa cells were either transfected with shRNA targeting *OAZ1* or control shRNA. As shown in Fig 1A, the level of *OAZ1* mRNA expression in granulosa cells transfected with shRNA *OAZ1* at 48 and 72 h was significantly lower than the control (*p<0*.*05*). The viability of granulosa cells transfected shRNA *OAZ1* at 48 h was significantly higher than the control (*p<0*.*05*) (Fig 1B). Thus, granulosa cells at 48 h after shRNA *OAZ1* transfection were employed to conduct the subsequent research.

### *OAZ1* knockdown elevated putrescine and spermidine contents by mediating the expression of polyamine metabolic enzyme genes

**Fig 2:**
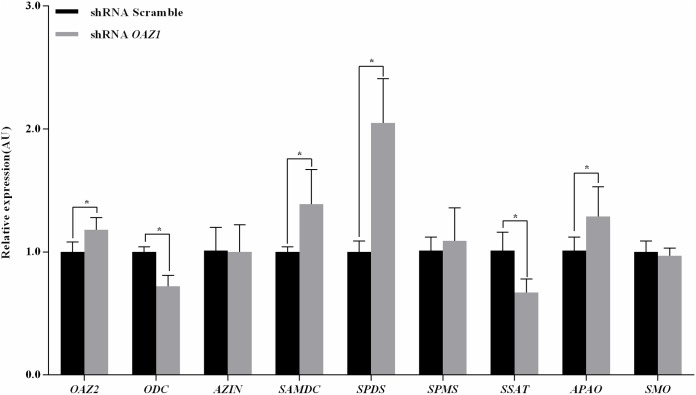
Expression levels of genes related to polyamine metabolism in granulosa cells. The data were presented as the mean ± SEM. Bars with a star were significantly different (p<0.05).

**Fig 3:**
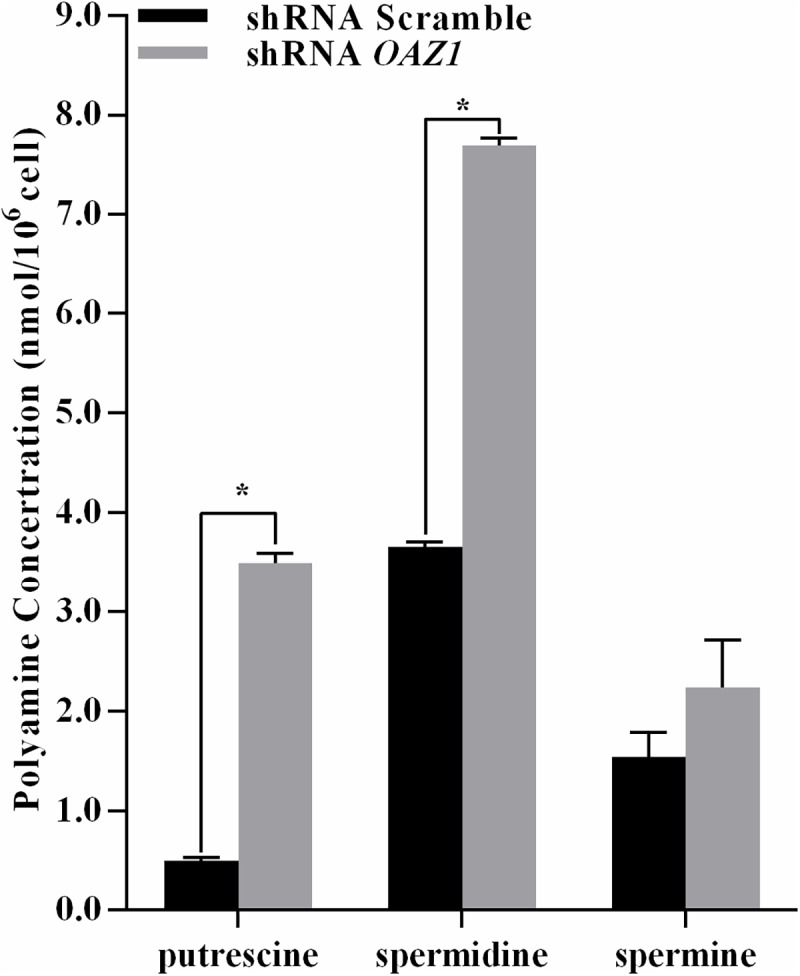
Polyamines concentration in granulosa cells. The data were presented as the mean ± SEM. Bars with a star were significantly different (p<0.05).

To determine the action of *OAZ1* regulating polyamine homeostasis in primary granulosa cells from geese, the expression levels of polyamine metabolic enzyme [3, 25, 26] genes and polyamine levels (Figs 2 and 3, and Table 2) were measured. The *OAZ2*, *SAMDC*, *SPDS*, and *APAO* mRNA expression levels in granulosa cells silencing *OAZ1* were significantly higher than the scramble (*p<0*.*05*). The *ODC* and *SSAT* mRNA expression levels in granulosa cells down-regulating *OAZ1* were significantly lower than the control (*p<0*.*05*). The putrescine and spermidine contents in granulosa cells transfected shRNA *OAZ1* were 7.04- and 2.11-fold higher compared with the control, respectively (*p<0*.*05*). However, spermine concentration was not significantly different compared with the control (*p>0*.*05*).

**Table 2:** Polyamines concentration (nmol/10^6^ cell) in granulosa cells.

	shRNA Scramble	shRNA OAZ1
Putrescine	0.49 ± 0.03	3.45±0.09*
Spermidine	3.65 ± 0.05	7.69±0.07*
Spermine	1.55 ± 0.24	2.24±0.47

The data were presented as the mean ± SEM.

*denotes a significantly different (p<0.05).

### *OAZ1* altered expression abundance of cell proliferation and apoptosis genes in granulosa cells

**Fig 4:**
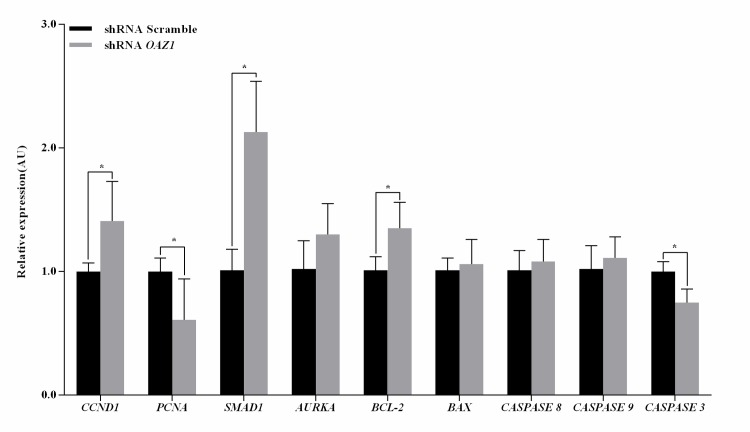
Expression levels of genes related to granulosa cell proliferation and apoptosis. The data were presented as the mean ± SEM. Bars with a star were significantly different (p<0.05).

Further, we performed qPCR to elucidate the effect of *OAZ1* knockdown on the transcriptions of genes related to granulosa cell proliferation and apoptosis. As shown in Fig 4, the amount of *CCND1* (encoding the Cyclin D1 protein which is crucial for the transition of cells from G1 to S phase), *SMAD1* (encoding the SMAD1 protein which is an intracellular protein that transduce extracellular signals from TGF beta ligands to the nucleus), and *BCL-2* (encoding the BCL-2 protein which is specifically considered an important anti-apoptotic protein) mRNA expression in granulosa cells down-regulating *OAZ1* was significantly higher than the control (*p<0*.*05*), and were 1.41-, 2.13- and 1.35-fold higher compared with the control, respectively. Whereas the *PCNA* (a cell proliferation marker) and *CASPASE 3* transcripts in granulosa cells down-regulating *OAZ1* were 0.61- and 0.75-fold higher, respectively, compared with the control (*p<0*.*05*). However, the expression levels of *AURKA* (encoding the AURKA protein which plays an essential role in mitotic events, peaks during the G2 phase to M phase transition), *BAX* (encoding the BAX protein which forms a heterodimer with BCL-2 and functions as an apoptotic activator), *CASPASE 8* and *9* were not significantly different than the vehicle group (*p>0*.*05*).

### *OAZ1* knockdown down-regulated estradiol concentration in culture media and ER and LHR mRNA expression levels in granulosa cells

**Fig 5:**
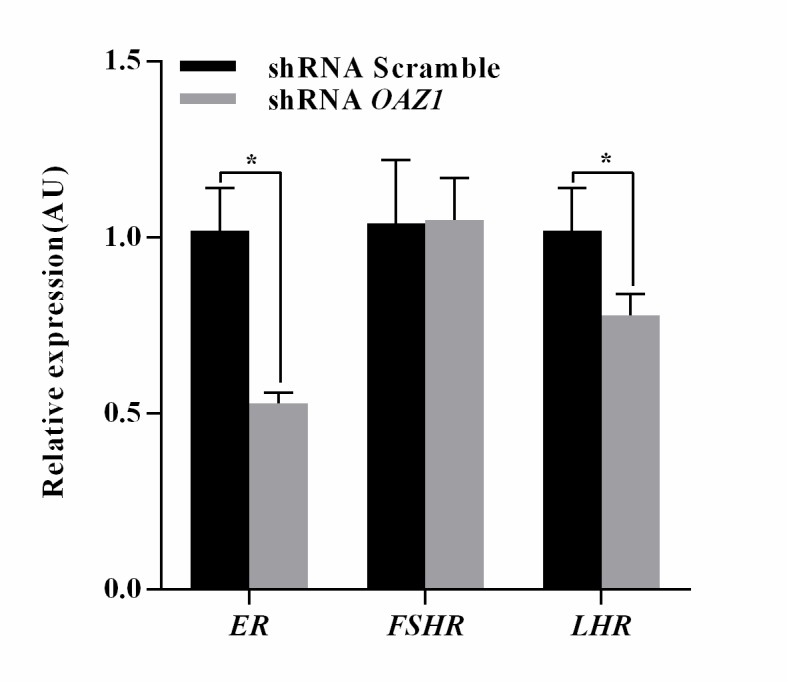
Expression levels of hormone receptor genes in granulosa cells. The data were presented as the mean ± SEM. Bars with a star were significantly different (p<0.05).

The estradiol concentration underwent a slight but statically significant decrease in culture media for granulosa cells down-regulating *OAZ1* compared with the control (748.73±4.21 *vs* 781.65±6.69 pg/ml) (*p<0*.*05*), while FSH and LH contents were not significantly different. As shown in Fig 5, the *ER* and *LHR* mRNA expression levels were significantly lower in granulosa cells silencing *OAZ1* compared with the control (*p<0*.*05*).

## Discussion

Numerous studies have found that polyamines play key roles in both normal and abnormal functions of cells [27]. OAZ1 plays a central role in the regulation of polyamine homeostasis [28]. Increased OAZ1 levels in response to polyamines led to inhibition of growth in IEC-6 cells, which was restored by inhibiting OAZ synthesis by asparagine in the presence of spermidine or spermine [29]. In agreement with that study, our result corroborated further that down-regulated *OAZ1* enhanced the growth of primary granulosa cells. Enhanced levels of polyamines, including putrescine and spermidine, are associated with cell proliferation and hyper-proliferation [30]. Furthermore, *OAZ1* affects cell proliferation and viability solely by modulating cellular polyamine metabolism in NIH3T3 and HEK-293 cells [31]. However, Ray *et al*. suggested that the effect of *OAZ1* on IEC-6 cell growth was independent of polyamines [32]. In the present study, *OAZ1* silencing resulted in an increase of the putrescine and spermidine concentrations, and an unchanged spermine concentration. This finding was in keeping with the result in HEK-CMV-Luc2-Hygro cells reported by Xiao *et al*. [33]. Thus, in consideration of the mechanism of polyamine action varying among cell types, whether increased viability of granulosa cells silenced *OAZ1* resulted from suppressed *OAZ1* expression, or decreased the putrescine and spermidine contents, remains to be determined.

*OAZ2* has structure and tissue distribution similar to that of *OAZ1*, but it is expressed at significantly lower levels [8]. *OAZ1* knockdown in granulosa cells induced *OAZ2* expression, suggesting that *OAZ2* played an important role in suppressing ODC by compensating for decreased *OAZ1* [8, 34]. Increased *OAZ2* and decreased *ODC* transcription might contribute to counteract sharp increase of the putrescine content in granulosa cells silencing *OAZ1*. It is possible that augmented spermidine content in granulosa cells down-regulating *OAZ1* resulted from enhanced levels of *SAMDC* and *SPDS* mRNA expression. These results suggested that *OAZ1* knockdown disturbed intracellular polyamine homeostasis with elevated putrescine and spermidine contents, and accelerated the growth of granulosa cells of geese *in vitro*.

OAZ1 has been shown to function by inhibiting cell proliferation and antitumor activity [14]. In the current study, *CCND1*, *SMAD1*, and *BCL-2* mRNA expression in granulosa cells down-regulating *OAZ1* with decreased *CASPASE 3* expression level were significantly higher than the scramble group. Polyamine biosynthesis has been shown to peak at the G1/S transition regulated by *CCND1* [35]. *OAZ1* overexpression in zinc-deficient mice restrained *CCND1* expression in the forestomach [14]. *SMAD1* is known to serve as a signaling intermediate for bone morphogenetic proteins and anti-mullerian hormone, which are critical in regulating granulosa cell growth and differentiation [36]. *OAZ1* overexpression induced a small decrease in the concentration of BCL-2 protein [37]. It corroborated the hypothesis that *OAZ1* functions as inhibiting cell proliferation and promoting apoptosis. These results, including our data, indicate that *OAZ1* knockdown enhanced granulosa cell growth by up-regulating the transcriptions of proliferation-promoting and anti-apoptosis genes, and suppressing the activation of apoptosomal *CASPASE 3* cascade. As mentioned above, OAZ1 is a negative regulator of cell proliferation [9, 10], and PCNA is a cell proliferation marker. Our present data showed that the viability of granulosa cells down-regulating *OAZ1* increased, while the level of *PCNA* mRNA expression decreased. However, the reason remains to be elucidated. Additionally, though OAZ1 can mediate degradation of AURKA [17], our study suggested that silencing of *OAZ1* did not alter *AURKA* transcription.

Numerous studies have suggested that FSH, LH, and estradiol induce ODC activity and hence polyamines [4, 38]. Bastida *et al*. reported that ODC/polyamines played important roles in mediating the effect of LH on follicular development and luteinization [39]. Polyamines are also important in directly regulating the ligand–membrane receptor interaction and gene-activating functions of *ER* in human breast cancer cells [40, 41]. Our results showed that knockdown of *OAZ1* decreased estradiol content in culture media and suppressed *ER* and *LHR* mRNA expression in granulosa cells of geese. On the contrary, *OAZ1* overexpression suppressed *ER* mRNA expression in human breast cancer cells [42]. One possible explanation was that the function of *OAZ1* mediating *ER* transcription might vary between primary normal and cancer cells. Additionally, oligoamines (a specific polyamine analogues) suppressed expression of *ER* in human breast cancer cells [43]. Whether increased putrescine and spermidine concentrations decreased *ER* mRNA expression level remains to be determined. Taken together, data from our study and others indicate that OAZ1 and/or polyamines play important roles in the responsiveness of granulosa cells toward estradiol and LH in geese, though a possible mechanism remains to be elucidated.

## Conclusions

In conclusion, *OAZ1* knockdown, with elevated putrescine and spermidine contents enhanced granulosa cell viability by affecting the transcriptions of genes related with granulosa cell proliferation and apoptosis, as well as inhibited *ER* and *LHR* transcriptions of granulosa cells in geese. Our current study provides the first evidence that OAZ1 and/or polyamines play an important role in regulating primary granulosa cell viability and hormone receptor expression.
